# Commentary: Cross-Cultural Comparison of Self-Construal and Well-Being between Japan and South Korea: The Role of Self-Focused and Other-Focused Relational Selves

**DOI:** 10.3389/fpsyg.2019.02885

**Published:** 2019-12-20

**Authors:** Rowena L. T. Kong

**Affiliations:** Department of Psychology, The University of British Columbia, Vancouver, BC, Canada

**Keywords:** self-construal, perception, cognition, interdependence, relation

## Introduction

The research article “Individual Self, Relational Self, Collective Self: Hierarchical Ordering of the Tripartite Self” authored by Sedikides et al. (2011) described clearly the differences between and implications of the three forms of distinct individual, relational, and collective selves. Based on this theoretical framework, Park et al. (2017) went further to define self-focused and other-focused relational selves. According to the authors, the self-focused relational self is a form of self-other interacting self which values other's impression and opinion of the self, but from the point of view of the self. In a way, such self is strongly introspective and bears a fundamental similarity with the individual self-due to the precedence of self-directed focus. On the other hand, the other-focused self is also relational but with the priority in focus placed on the other. What is uncertain and less well-defined is the level of self-influence on such a point of view, which is left to guess whether it is fully originating from other or simply the self-perceiving about other. Based on such conjecture, it is more realistic to assume that the focus on other in other-focused relational self can never possess a point of view that is purely and entirely other-originated. Under such circumstance, certain elements contributed by the self is necessary. This speaks to a limited discussion by Park et al. (2017) on the extent to which self-focused and other-focused relational selves could be less distinct or distinguishable from the individual self. While Sedikides et al. (2011) has highlighted the distinction between individual, relational, and collective selves based on the theoretical framework of independent and interdependent self-construals, such distinct definitions and concepts also appear to magnify the theoretical approach of compartmentalised perception and cognition. These view and approach bear solid boundary of differences between hierarchical forms of self and are less flexible to shared characteristics. There are many parallel processes taking place constantly in the human brain that aid our ability of social cognition and consequently contribute to our framework of understanding and assessment of both differences and similarities between entities and concepts. Park et al. (2017) were focused and clear on defining the distinctions between self-focused and other-focused selves, with less weight of discussion given to their underlying shared characteristic(s). Therefore, a more conceptually rich and balanced interpretation that links our cognition and concept of self-construals is needed. A suggested factor that takes into account self-other relative priority that sources the origin of direction of one's perception offers a less discrete view of the tripartite self and sub-relational self. This seamlessly incorporates a dimension of both shared similarities and differences which interact dynamically with each other.

## Factor of Direction in Self-Other Perception

A factor that could potentially link the three types of individual self, relational self, and to a lesser degree, the collective self, helps complement the heavy focus by the above authors on differentiating and drawing sharp definition boundaries between these categories and subcategories of the construed self. In deciding the candidate for position of priority in originating the direction of one's perception and cognition, it sets the stage for contention between the self and other in directing attention on whether it is “self toward other” or “other toward self.” The choice between this pair of directional opposites which influences the point of origin of our attention, perception, and cognition could take into consideration and vary based on situational and cumulative level of position importance or significance in role and identity between self and other as evaluated by the self. For example, a mother with a higher authority of parental role and identity based on self-other awareness, knowledge and both past and present interactions with her daughter would provoke a greater likelihood of the latter's “other toward self” perception. On the other hand, a “self toward other” direction in attention may hold when the daughter is addressing her younger sibling due to the shift in age and role precedence from an older mother to a younger member in the family. This is based on the notion that we can perceive our self or other(s) as occupying a level of equal or a different higher or lower importance.

## Discussion

Intuitively, the other-focused relational self who tends to perceive the self from the point of view of other may assume an “other toward self” direction of perception or “facing” the self. In this case, this individual could place the self at a position of lower importance relative to other in comparison to the individual and self-focused relational selves, as the self-values more of the role and opinion of other. For example, an individual who admires the greater academic achievements of a friend or classmate would perceive the friend more than the friend perceives the self, i.e., the friend faces the self within the individual's introspective imagery as a result of respect for her superior school performance. Therefore, the boundary lessens between the individual self and self-focused relational self when these two modestly distinct forms of self converge in terms of both origin and direction of focus or target of perception, as emphasised by Park et al. (2017). Such perception originates from the self and yet, is simultaneously directed toward the self as the target, leading to a common ground from which processing of cognition and judgement(s) emerge. Whereas, the definition for individual self by Sedikides et al. (2011) is more encompassing, covering perception of both self and other(s), the less solidly defined subcategories of self-focused and other-focused relational selves by Park et al. (2017) may lend to implicit similarities between self-focused and individual selves because both assume self-directed and targeted self-perception. The point of convergence is therefore, the self, whether it is the source or target, because a relational self is simply an aid in explaining the theme of action and exchange between more than one involved participants.

In conclusion, our perception and cognition are highly dynamic parallel processing that varies according to content and context. They work constantly in multi-functional mode as opposed to predictable serialised processing (Evans, 2008; Behrens et al., 2009). Therefore, it is best that research goes beyond the limitations of unifocaled and distinction-oriented theories to capture their unbounded versatility. Perhaps, the long-standing tradition of Western research which originates from individualistic approach has prioritised distinct and discrete characteristics of theoretical elements over shared and/or mutual similarities. Research outcomes and interpretations tend to assume that an individual's perception of the level of importance and stature of other(s) in relation to self maintains constancy for a considerable duration of time. Practical real-life experiences may speak to the contrary. To the extent that self-other interactions are liable to conflicts and disagreements on values and opinions, one's perception of and respect for another may change over time, leading to a fluid displacement of their role of importance in our lives and mindsets. In diverse ethnic communities, this form of self-other dynamics may be even more pronounced. In light of this article, if the factor of self-other relative position of importance in originating the directional focus of our attention and perception is utilised to assess its influence on the three forms of individual, relational and collective selves, future studies could potentially help better define their similarities and common grounds and not merely in terms of their discrete properties.

## Author Contributions

RK contributed to the idea and concept of article content.

### Conflict of Interest

The author declares that the research was conducted in the absence of any commercial or financial relationships that could be construed as a potential conflict of interest.

## References

[B1] Behrens T. E. Hunt L. T. Rushworth M. F. (2009). The computation of social behavior. Science 324, 1160–1164. 10.1126/science.1169694 19478175

[B2] Evans J. S. B. (2008). Dual-processing accounts of reasoning, judgment, and social cognition. Annu. Rev. Psychol. 59, 255–278. 10.1146/annurev.psych.59.103006.093629 18154502

[B3] Park J. Norasakkunkit V. Kashima Y. (2017). Cross-cultural comparison of self-construal and well-being between Japan and South Korea: the role of self-focused and other-focused relational selves. Front. Psychol. 8:1516. 10.3389/fpsyg.2017.01516 28928699 PMC5591841

[B4] Sedikides C. Gaertner L. O'Mara E. M. (2011). Individual self, relational self, collective self: Hierarchical ordering of the tripartite self. Psychol. Stud. 56, 98–107. 10.1007/s12646-011-0059-0

